# The Effects of Elevated Specific Conductivity on the Chronic Toxicity of Mining Influenced Streams Using *Ceriodaphnia dubia*

**DOI:** 10.1371/journal.pone.0165683

**Published:** 2016-11-04

**Authors:** Mindy Yeager Armstead, Leah Bitzer-Creathers, Mandee Wilson

**Affiliations:** 1 Department of Integrated Science & Technology, Marshall University, Huntington, West Virginia, United States of America; 2 Potesta & Associates, Inc., Charleston, West Virginia, United States of America; Chinese Research Academy of Environmental Sciences, CHINA

## Abstract

Salinization of freshwater ecosystems as a result of human activities has markedly increased in recent years. Much attention is currently directed at evaluating the effects of increased salinity on freshwater biota. In the Central Appalachian region of the eastern United States, specific conductance from alkaline discharges associated with mountain top mining practices has been implicated in macroinvertebrate community declines in streams receiving coal mining discharges. Whole effluent toxicity testing of receiving stream water was used to test the hypothesis that mine discharges are toxic to laboratory test organisms and further, that toxicity is related to ionic concentrations as indicated by conductivity. Chronic toxicity testing using *Ceriodaphnia dubia* was conducted by contract laboratories at 72 sites with a total of 129 tests over a 3.5 year period. The database was evaluated to determine the ionic composition of mine effluent dominated streams and whether discharge constituents were related to toxicity in *C*. *dubia*. As expected, sulfate was found to be the dominant anion in streams receiving mining discharges with bicarbonate variable and sometimes a substantial component of the dissolved solids. Overall, the temporal variability in conductance was low at each site which would indicate fairly stable water quality conditions. Results of the toxicity tests show no relationship between conductance and survival of *C*. *dubia* in the mining influenced streams with the traditional toxicity test endpoints. However, consideration of the entire dataset revealed a significant inverse relationship between conductivity and neonate production. While conductivity explained very little of the high variability in the offspring production (r^2^ = 0.1304), the average numbers of offspring were consistently less than 20 neonates at the highest conductivities.

## Introduction

Secondary salinization of freshwater ecosystems can result from a broad array of human activities such as irrigation, mining, and de-icing activities [1]. With demands on limited aquatic resources increasing, much attention is currently directed at evaluating the effects of salinization on freshwater biota. The effects of acid mine drainage on aquatic life in streams of the Central Appalachian region of the eastern United States have long been studied. Recent attention has turned to the effects of alkaline discharge associated with mountain top mining practices in the Central Appalachian coalfield region [2]. Alkaline discharges are comprised mainly of dissolved solids produced from weathering of fractured rock in overburden generated during the mining process with values to 3,700 μS/cm reported [3, 4, 5, 6]. Increased ionic concentrations can be widespread in mining influenced streams as entire watersheds are affected minimizing the potential for dilution. Discharges from the toe of a valley fill may comprise the entire stream flow making the discharge and the stream water one in the same for some distance below the fill. Often the discharges are not diluted for some distance as even confluence with another discharge-dominated tributary will serve to increase or maintain conductivity levels [5]. The increased dissolved ions have been suggested as contributors to impairment of benthic macroinvertebrate communities and declining mayfly (Order Ephemeroptera) populations in streams receiving mining discharges [7]. Physical effects, such as excess fine sediment, which might be increased in streams downstream of valley fills [8] or turbidity from soil erosion, accumulation of coal fines, and smothering of the stream substrate from precipitated metal compounds, may also occur [9, 10].

Dissolved solids are constituents of natural waters necessary for supporting aquatic assemblages. Some dissolved ions directly support the organisms, such as macro-minerals potassium and magnesium [11], or chloride which is important for osmoregulation [12]. Other ions are important in the organism’s external environment such as the bicarbonate ion which provides buffering, or calcium and magnesium which mitigate metal toxicity [13]. In freshwater ecosystems of the Appalachian mining region, ionic constituents are primarily sodium, calcium, magnesium, potassium, chloride, sulfate and bicarbonate [7, 14]. Much is known about the toxicity of dissolved solids individually and in complex mixtures. Toxicity can occur if ion concentrations are too low or too high for aquatic organisms to osmoregulate properly [15]. Toxicity is hard to predict from general estimators like total dissolved solids (TDS) or specific conductance (hereafter referred to as conductivity) measurements due to the variable toxicity of individual ions that comprise these total measures. Mount and others [14] established the relative acute toxicity of seven major ions to three freshwater organisms and developed statistical toxicity models to predict the acute toxicity of complex ionic mixtures. The relative toxicity level of sulfate, the dominant ion in receiving streams of mining effluent, is low compared to the other ions tested [14]. There is also much evidence that the presence of two or more ions can ameliorate toxicity and result in lower toxicity levels than expected by individual ions [14, 16, 17, 18]. In particular, hardness is known to mitigate sulfate and chloride toxicity [16, 17, 19], but does not affect the toxicity of the bicarbonate ion [20]. The chronic toxicity of complex salt mixtures is also well studied and the acute to chronic ratios of salts are relatively low [12, 20, 21].

Conductivity is often used as a surrogate for disturbance [22] and is correlated with urban development and agriculture [23]. Conductivity is also correlated with coal mining activity in the Appalachian region [5, 7] and has been implicated as a causative factor impairing macroinvertebrate communities, and specifically mayflies (Ephemeroptera), in mining regions [24, 25]. However, because of the variable toxicity of individual ions and complex mixtures, the use of indicator parameters, such as TDS and conductivity for the protection of aquatic life is of questionable value [17]. Toxicity testing of complex mixtures and effluents is necessary to determine the relative toxicity of specific waters.

Recent studies have suggested that results of laboratory toxicity testing may not adequately characterize field effects of elevated dissolved solids [24, 25] and other contaminants such as metals [26] and pesticides [27]. Empirical evidence supporting measured exposures and quantitative measures of regional biodiversity may be lacking [27]. Two studies which do address this linkage focus on salinity effects on macroinvertebrate assemblages in Australia [28, 29] where the relationship between laboratory testing and macroinvertebrate sensitivity was previously established [30]. The discrepancy between field-derived impairment thresholds of sulfate-dominated mining discharges [25], and the reported laboratory toxicity values of salt mixtures described above, have prompted interest in further defining the link between the measured field concentrations and biodiversity declines. One hindrance to establishing the relationship between in-stream effects of mining discharges and native taxa are the difficulties in obtaining sensitive life stages of the native taxa for use in toxicity testing. Currently, the risks to aquatic environments are primarily described by a few species of model organisms, such as *Ceriodaphnia dubia*, with the relationship between these organisms and native taxa currently under investigation [31].

Whole effluent toxicity (WET) testing has been used as part of the National Pollutant Discharge Elimination System (NPDES) permitting process to evaluate complex effluents, specifically those with potential toxicity not protected by specific numeric criteria. WET testing has recently been employed by contract laboratories to evaluate toxicity in streams receiving high conductivity discharges in mining regions of West Virginia. The WET testing was conducted to test the hypothesis that water in streams receiving mine discharges are toxic to laboratory test organisms, and further, that toxicity is related to ionic concentrations as indicated by conductivity. The specific objectives of the study herein were to examine the existing water chemistry data to determine the ionic composition of mine effluent dominated receiving streams in the region, to evaluate the available toxicity testing database in order to establish whether stream waters receiving mining discharges are toxic to the standard toxicity text organism, *C*. *dubia*, and, if toxicity is found, to relate toxicity to discharge constituents, specifically those contributing to overall conductivity or TDS measurements.

## Methods

### Sampling Site Selection

**Fig 1:**
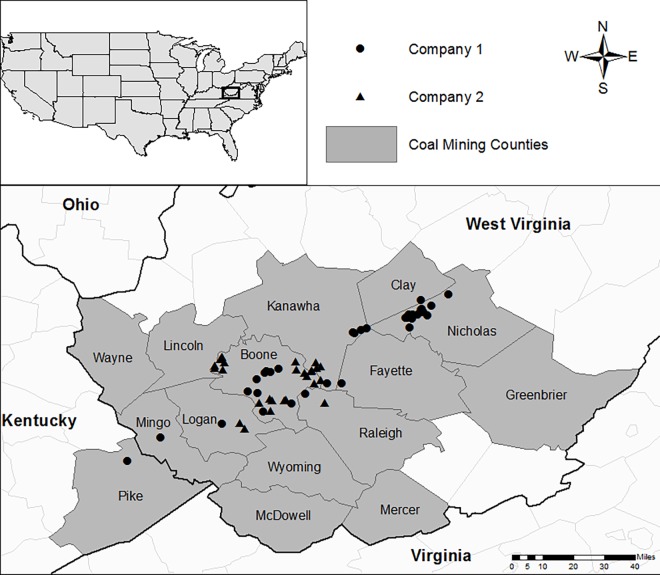
Sampling locations in the southern West Virginia mining region. One sampling location was located in Kentucky with the stream originating in West Virginia.

Two coal companies operating in West Virginia were required to conduct semi-annual WET testing in receiving streams where conductivity values greater than 1,500 μS/cm had been recorded in monthly discharge monitoring. Each company operates multiple facilities in the region with discharges characteristic of alkaline mine drainage that were evaluated for sampling. Each year the testing was required at 10 sampling locations for each company which were pre-approved by the United States Environmental Protection Agency (USEPA) and subsequently sampled for testing under low-flow (summer/fall) and high-flow (winter/spring) conditions. Sampling was initiated in December of 2008 and continued to November 2011 with a total of 129 chronic *C*. *dubia* toxicity tests presented from a total of 72 stream sampling locations (Fig 1). Samples were collected at the permitted in-stream sampling location where conductivity values greater than 1,500 μS/cm had been recorded in monthly discharge monitoring. Each of these in-stream sampling points was located downstream of an NPDES permitted outfall from a surface or deep mine which was compliant with permit conditions. During the first round of testing, seasonal duplication was not required. Subsequently, seasonal testing was successfully implemented at 57 of the 72 sampling locations. Specific permission for sampling activities pertaining to this study was not required because sampling was conducted by the landowner or their designated contractors. The field studies did not involve endangered or protect species, or vertebrates.

### Water Chemistry Analysis

Field water quality measurements were taken at the time of sample collection by both companies. At 28 of the 72 sampling stations, field parameters measured included conductivity, pH, temperature, and dissolved oxygen (DO) which were recorded by a YSI 556 multi-parameter meter. At 44 stations, DO was not recorded and conductivity, pH and temperature were recorded using an Orion dual parameter meter. Both meters were calibrated daily per the manufacturers’ instructions on all sampling dates. Beginning in 2011, additional laboratory analysis was conducted on grab samples collected at one company’s 10 annual sampling locations. This analysis included the following parameters: total alkalinity, total sulfates, total chloride, total sodium, total magnesium, and total calcium. Water samples were grab samples collected mid-channel, mid-depth in clean, sampling containers provided by the laboratory. Nalgene sampling containers and preservative, if preservation was required, were appropriate for the analysis being conducted. Analyses were conducted at a West Virginia State Certified analytical laboratory using approved methodologies. The analytical methods included ion chromatography (EPA300.00) for sulfates and chlorides, and Inductively Coupled Plasma-Atomic Spectrometry (EPA200.7) for other ions. Total alkalinity and hardness were analyzed using methods 2320B and 2340B from Standard Methods 20^th^ edition [32].

### Toxicity Testing

Chronic toxicity testing, which followed standardized USEPA Method 1002.0, is briefly described herein. Water for toxicity testing was collected three times during the course of the seven-day tests [11]. Water was collected, to the extent practical, from mid-channel, mid-depth locations in dedicated 3.78 liter containers and stored in coolers on ice during transport to the laboratory. Samples were transported to the local laboratory on the day of collection when tested locally and were shipped overnight to out of state laboratories. Each test consisted of 5 serial dilutions utilizing high conductivity stream water as the test solution (100%) and USEPA moderately hard reconstituted water as the diluent and control. New test solutions were prepared daily and the organisms were transferred to clean test chambers. Organisms utilized in the seven-day static renewal tests were less than 24-hours old at the time of test initiation. Organisms were fed *Selenastrum capricornutum* concentrate and yeast, cereal leaf and trout chow suspension, each at a rate of 0.1 mL per 15 mL of test solution daily. Both food items were prepared per the USEPA method [11]. Tests were maintained at 25±1°C under ambient lighting on a 16-hour light and 8-hour dark daily cycle. Dissolved oxygen was maintained at greater than 4 mg/l oxygen and pH was within the 6 to 9 SU range. Any deviations from the required conditions are noted below. Mortality and neonate production were noted daily in the test chambers at the time of water renewal. Endpoints evaluated included survival and reproduction. Each test met the requirement of 80% or greater control survival and greater than 15 offspring per surviving female. Reproductive output in the control organisms averaged 20.5 neonates and ranged from 16.1 to 28.3.

While multiple laboratories were used in testing, all the testing was conducted in accordance with the US EPA methods at laboratories certified by the National Environmental Laboratory Accreditation Conference (NELAC) to ensure the quality of data obtained at the different laboratories. Three levels of quality assurance are therefore incorporated into the testing: each test met minimum requirements of control survival and neonate production; each laboratory had a quality assurance/quality control program that included reference toxicant testing to monitor the health of the organisms; each laboratory met the requirements for NELAC certification which included review and demonstration of organism fitness, staff training and compliance with methodology. All tests considered in the evaluation met quality assurance/quality controls requirements except for two discrepancies. In one test conducted in February 2011, the pH of the 100% and 50% test solutions drifted upward of 9.0 Standard Units (SU) during the exposure period. While initial values were below 9.0 SU, final pH values in these concentrations ranged up to 9.35 SU in the last 4 days of the exposure period. Additionally, while water for toxicity testing is to be collected three times during the course of the seven-day tests, an exception to this occurred in tests conducted during February 2010. During this testing period inclement weather prevented the collection of the second sample at the five sampling locations. Water outside of the holding time (from initial sampling) was used for test change-over in the five tests which were conducted in this time period. These deviations were considered minimal and the data were not excluded from the analysis. No other deviations from acceptable protocols were noted in the tests.

### Data Evaluation

Field collected water chemistry values for dissolved oxygen and pH were evaluated for consistency with applicable water quality criteria in West Virginia streams. These regulations indicate that dissolved oxygen values should be greater than 5.0 mg/l and pH values should be between 6 SU and 9 SU for the protection of warm water aquatic life. Variability of specific conductance within samples conducted for each test and between high- and low-flow sampling events is presented graphically. The relative change between high- and low-flow tests was determined by calculating the percent change of TDS (TDS = 0.65*specific conductivity) between sampling events. This difference is also presented graphically.

Statistical evaluations of toxicity testing were conducted by the laboratories performing the tests in accordance with the USEPA methods [11]. Test endpoints evaluated included the 25% inhibition concentration (IC25), the no observable effects concentration (NOEC), lowest observable effects concentration (LOEC), and the percent survival in 100% stream water. The IC25 represents the test concentration where neonate production was reduced by 25% and was calculated using the linear interpolation method, a point estimate technique [11]. The NOEC and LOEC were calculated by hypothesis techniques which included the T-test with Bonferroni Adjustment, Dunnett’s Test, Steel’s Many-one Rank Test, and Wilcoxon Rank Sum Test with Bonferroni Adjustment, depending on whether the data meet assumptions appropriate for each test [11]. The relationship between conductivity and the toxicity test endpoints of survival (as percent mortality) and reproduction (IC25) were to be evaluated using regression techniques; however the high number of tests which failed to generate toxicity resulted in a dataset which was not conducive to evaluation by either parametric or non-parametric regression techniques. The data were not normally distributed and the high number of ties in the dataset prohibited the use of ranking procedures. Thus, the data are presented graphically. Changes in conductivity over the test duration were evaluated for correlation to *C*. *dubia* impairment, as indicated by IC25 values, and is also presented graphically due to the high number of tests which generated no impairment. The relationship between conductivity and neonate production in the 100% stream water concentration was evaluated by linear regression and an analysis of variance procedure testing whether the slope of the regression line equaled zero. Comparison of the average conductivity values from the NOEC concentration in the 129 tests was conducted by one-way analysis of variance procedures followed by Tukey-Kramer multiple comparison tests to look for significant differences.

## Results

**Fig 2:**
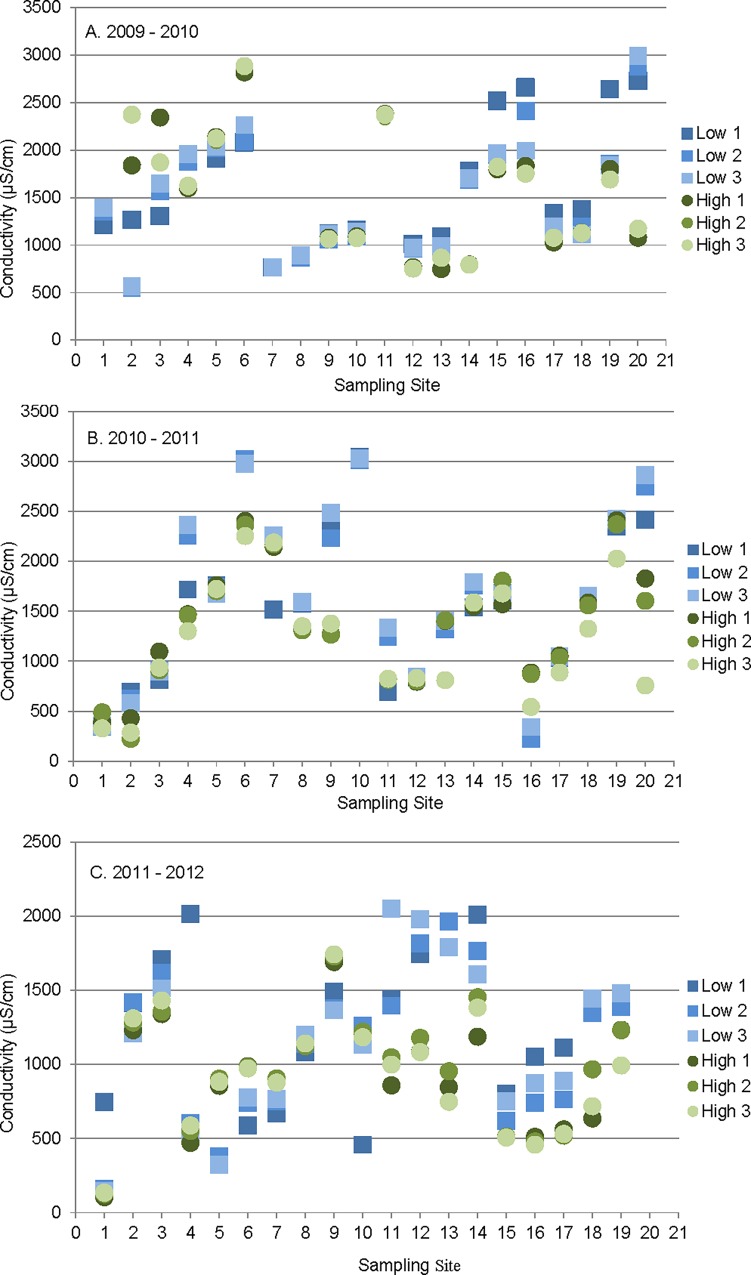
**A-C. Conductivity of samples.** Specific conductance of 3 samples collected from each site over 7-day toxicity tests conducted under low flow (blue square) and high flow (green circle) conditions during 2009–2010 (A), 2010–2011 (B), and 2011–2012 (C).

Water chemistry evaluations indicated that, with few exceptions, sampling sites were within a pH range of 6 to 9 SU at the time of sampling. The range of DO was generally from 8.0 mg/l to 12.0 mg/l, with few exceptions. Two sampling sites in the 2010 low-flow sampling event had low field DO readings, ranging from 4 to 5 mg/l, however, acceptable DO concentrations were maintained in the laboratory testing. Temperature was variable but no exceptional values were noted for the sampling conditions. Conductivity values ranged from 68 μS/cm to 3047 μS/cm at the 72 sites during the testing (Fig 2A–2C).

**Fig 3:**
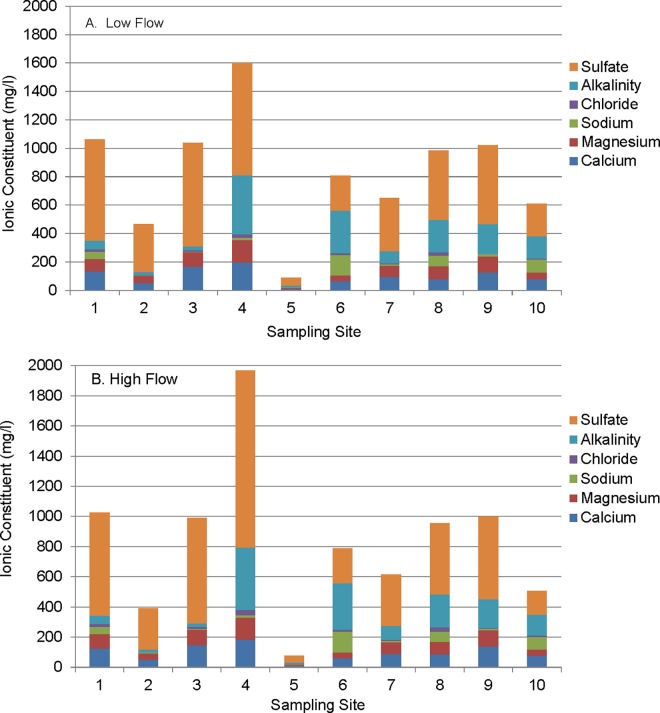
**A-B. Ionic Constituents.** Concentrations of ionic constituents in samples collected from ten sites during 2011 under (A) low flow (October and November) and (B) high flow (May and June) conditions.

Laboratory analyses conducted on samples collected from 10 sites in 2011 provide relative proportions of the ionic constituent (Fig 3A and 3B). Anion concentrations were dominated by sulfate, followed by bicarbonate (measured as alkalinity) and, to a lesser extent, chloride. The cations calcium, magnesium, and sodium balanced the ionic concentrations. Changes in the relative constituent concentrations between low-flow and high-flow sampling events provide information on dilution from surface flow. Decreases in specific ion concentrations in high-flow as compared to low-flow sampling indicate dilution by surface water. While 7 of the 10 sites showed increase in chloride from surface run off, only 1 of the 10 sites indicated substantial surface contributions of ions.

Conductivity values were compared from the sampling sites for which repeated testing was conducted during the three year periods (Fig 2A–2C). At many sites, there was little variability in conductivity values on the three collection dates during the 7-day test period. Also, many sites had little seasonal variability as indicated by variability between sampling events. In the 2009 to 2010 sampling year Sites 8, 20 and 14 showed substantially lower conductivity under high-flow conditions indicating the effects of dilution. Site 2 showed higher conductivity under high-flow conditions potentially indicating a surface water source of dissolved solids. Other sites were generally similar between the sampling events. During the 2010 to 2011 testing period sites 2, 9, 16 and 20 showed the greatest relative variability. Overall, the greatest variability was seen during the 2011 to 2012 testing period. Most sites exhibited highest conductivity under low-flow conditions with the exceptions of Sites 2, 3, 6 and11 in 2009–2010, Site 16 in 2010–2011, and Sites 5, 6, 7 and 9 in 2011–2012.

**Fig 4:**
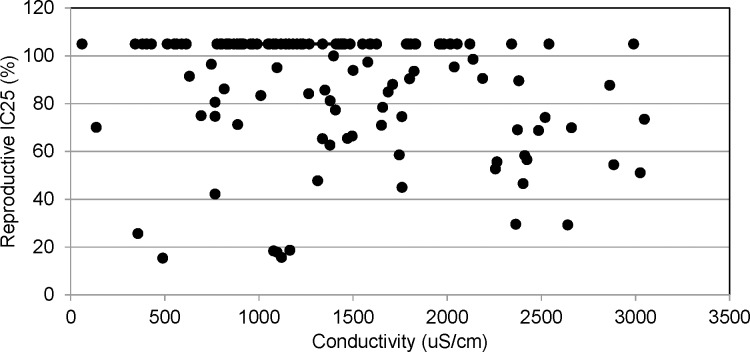
IC25 and conductivity. Reproductive endpoint (IC25) compared to the difference between maximum and minimum conductivities of samples utilized for toxicity testing.

The changing conductivity throughout each test did not appear to affect the test outcome as indicated by comparison of inhibition concentrations for reproduction (IC25) with the magnitude of conductivity differential imparted by the 3 water samples utilized during the test (calculated as difference of maximum and minimum conductivity) (Fig 4).

**Fig 5:**
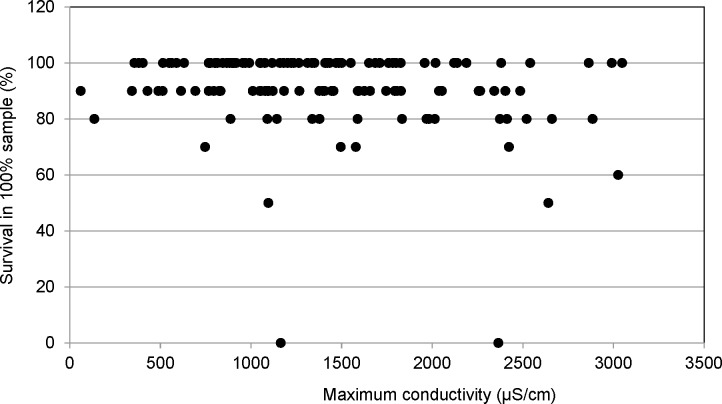
Survival in maximum conductivity. Plot of no observable effects concentration (NOEC) for survival versus maximum conductivity. The *C*. *dubia* percent survival in undiluted stream water showed no apparent relationship between conductivity and mortality within the aforementioned conductivity ranges.

Of the 129 tests conducted, 104 had survival of 90% or greater. Only nine tests exhibited survival less than 80% in the test organisms exposed to 100% stream water and only seven of the tests generated a NOEC less than 100% for survival. Maximum conductivity at the seven sites with substantial mortality ranged from 747 μS/cm to 3025 μS/cm. Overall, there was no apparent relationship between conductivity and mortality (Fig 5).

**Fig 6:**
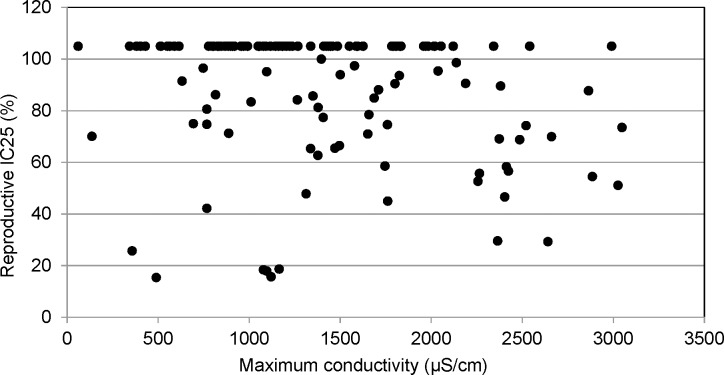
Reproductive endpoint, IC25, and maximum conductivity. IC25s greater than 100% are graphed as 105%. No apparent relationship was seen between maximum conductivity concentrations and *C*. *dubia* reproduction.

**Fig 7:**
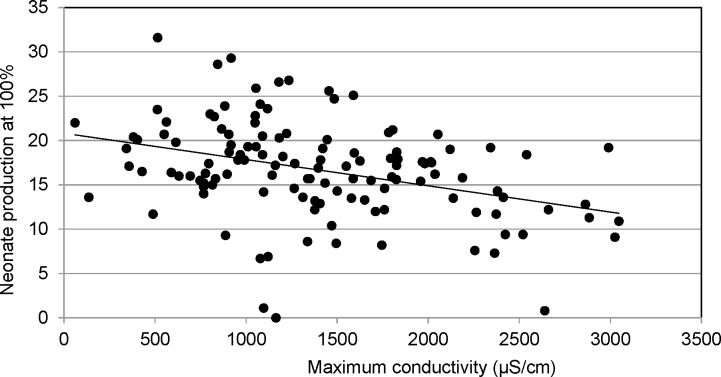
Neonate production and maximum conductivity. Regression of maximum conductivity and number of neonates per adult exposed to 100% samples (r^2^ = 0.1304, F = 18.45, p<0.001). Neonate production was generally less than 20 at conductivity levels greater than 2000 μS/cm.

**Fig 8:**
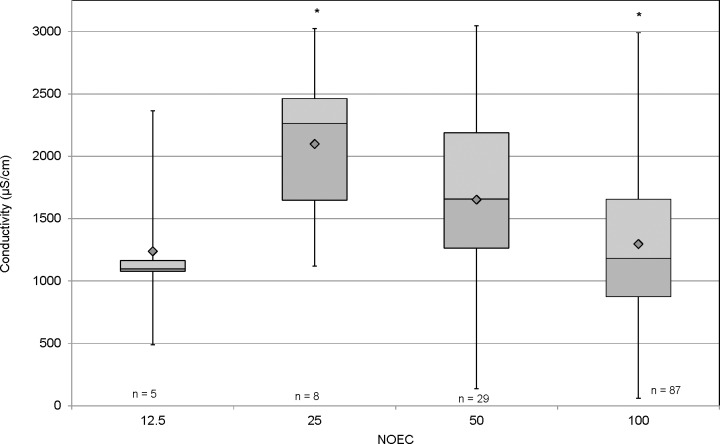
Means and ranges of maximum conductivity among samples grouped by NOEC. Asterisks indicate groups with significant different mean conductivity (p<0.05). A significant difference was found between the average conductivity of tests with no toxicity demonstrated (NOEC of 100%) and those generating an NOEC of 25% (F = 5.5; p<0.05).

The reproductive endpoint was more sensitive than survival in the chronic toxicity tests; although two thirds of the tests failed to generate an LOEC for reproductive impairment. A NOEC of less than 100% was determined in 42 of the tests with 29 having an NOEC of 50%, eight having an NOEC of 25% and five having an NOEC of 12.5%. There was no apparent relationship between maximum conductivity and reproductive impairment (IC25) demonstrated in the range of conductivities tested (Fig 6). However, linear regression analysis of neonate production in the 100% samples with maximum test conductivity showed a significant inverse relationship (r^2^ = 0.1304, F = 18.45, P<0.001). Neonate production was generally less than 20 at conductivity levels greater than 2000 μS/cm (Fig 7). While a correlation could not be established with the NOEC endpoint in an alternative analysis, higher conductivities were associated with lower NOEC values (Fig 8). A significant difference was found between the average conductivity of tests with no toxicity demonstrated (NOEC of 100%) and those generating an NOEC of 25% (F = 5.5; p<0.05). The average conductivity of tests with an NOEC of 50% was intermediate, but not significantly different. The average conductivity in the few tests with an NOEC of 12.5% was not dissimilar to those which generated no toxicity. This likely indicates that factors not associated with dissolved solids were responsible for toxicity in these tests.

## Discussion

Water chemistry parameters such as pH, DO and temperature are broad measures of stream condition. These parameters were found to be within acceptable ranges for supporting healthy aquatic communities in the present study. However, conductivity varied greatly among study sites as expected [5, 7]. This study was undertaken to characterize the ionic composition of streams receiving mining discharges and to evaluate potential toxicity in streams receiving mining discharges. The organism used in the testing was *C*. *dubia*, a laboratory-reared organism often used to predict in-stream effects of complex effluents and a widely used test surrogate for the protection of aquatic organisms [11, 31]. As indicated previously, the relative sensitivity of these model organisms with native taxa is under investigation by others [31] and in our laboratory; however, with the mining companies required to conduct the *C*. *dubia* testing, it is worthwhile to examine the extent of toxicity to this surrogate organism.

One potential risk to aquatic organisms exposed to high conductivity stream waters is that fluctuations in levels of dissolved solids require organisms to exert energy in maintaining osmoregulatory balance. This effort may be stressful for aquatic organisms and result in impairment. Overall, there was low variability in conductivity measured in the 3 samples collected to initiate and maintain the 7-day tests. At many sites there was surprisingly little variability in conductivity evident between the sampling seasons indicating the dominance of mine discharges and the lack of substantial surface flow. The few exceptions demonstrating within season variability (i.e. substantial changes during the test) may result from rain events during testing in the effluent dominated streams. Substantial variability between seasons indicated dilution of the low-flow condition shown as dips in the relative percent line or lower conductivity at high-flow. Alternately, there were three instances of substantial surface contributions of dissolved solids indicated as peaks or higher conductivity at high flows.

The finding of little variability in conductivity from low- and high-flow sampling depicts effluent-dominated or groundwater-dominated streams with limited surface inputs. Low-flow critical condition, or higher conductivity under low-flow conditions, is expected in streams generally offering dilution of the discharges at some point in the year (Fig 2 (a) Sites 16 and 19, (b) Sites 4 and 19, and (c) Site 4) while higher conductivity under high-flow may indicate non-point sources such as run-off as a primary contributor of ionic constituents. Based on these observations, non-point contributions do not appear to substantially influence conductivity. In other words, stream conductivity is not driven by storm water inputs in most of the streams evaluated. Although laboratory analyses were not conducted with each toxicity test, the 10 sites for which data are available generally support this “seasonal stability” in specific ionic constituents. Changes in individual ionic constituents were generally small and slight decreases under higher flow conditions indicated little dilution from surface water.

The mining influenced streams evaluated did not consistently demonstrate toxicity correlated with the ionic concentration when evaluated with respect to traditional toxicity testing endpoints such as NOEC/LOEC and IC25 in the 129 tests evaluated. Generally, the relationship between stressors and test organisms can be established by the dose response in the dilution series when the toxic effect is strong. That was not the case in the series of tests examined herein where toxicity was inconsistent or absent. However, when the relationship between increased conductivity and reduced number of offspring was evaluated for the larger dataset, it was significant. It was evident that neonate production was reduced at the highest conductivities. These findings may indicate that the conductivity tested in the current evaluation is approaching levels where significant effects would be demonstrated. The lack of substantial response at the levels represented likely results from the contribution of multiple ions, the dominance of the sulfate ion, and the relative contributions of calcium and magnesium, all factors which mitigate the toxicity of elevated dissolved solids to *C*. *dubia*. The presence of multiple salts results in lower toxicity than individual salts tested alone [14, 15, 17]. Hardness values in the range of 299 to 1133 mg/l CaCO_3_ would substantially mitigate toxicity of elevated dissolved solids, particularly sulfate, which was the dominant ion [33]. It is also noteworthy that conductivity explained very little of the high variability in the offspring production (r^2^ = 0.1304). Other water chemistry variables or mitigating factors may also be contributing to this variability.

Results of the 129 toxicity tests described herein are consistent with available literature concerning toxicity of sulfate-dominated mining effluents [34], simulated mining effluents [35] and sulfate toxicity [19, 20]. Kunz and others [36] reported the chronic toxicity of reconstituted waters simulating alkaline mine discharges to *C*. *dubia*. Similar to our findings, they reported impairment in 2 of the 3 waters tested with conductivities in the 1800 μS/cm to 2400 μS/cm range but no effects to the *C*. *dubia* in the third water tested which had a conductivity of approximately 1900 μS/cm. Kennedy and others [34] described the NOEC and LOEC of a sulfate dominated mining effluent as 2,910±40 and 3,710±46 μS/cm, respectively. In subsequent testing with simulated effluents, Kennedy and others [35] found chronic toxicity in sodium- and sulfate-dominated waters occurred at approximately 3200 μS/cm (LOEC and IC25). These findings are similar to our results which did not show consistent toxicity at conductivities below 3000 μS/cm.

Using the sulfate concentrations from the specific ion analysis presented in Fig 3, the relationship between conductivity and sulfate is sufficiently demonstrated (r^2^ = 0.8; p<0.001) that sulfate concentrations can be predicted from conductivity on dates the parameter was not specifically measured. This estimate shows average sulfate concentrations of approximately 1200 to 1500 mg/l in the conductivity range of 2000 μS/cm to 2500 μS/cm, which were the highest conductivities tested. Elphick and others [19] found NOEC/LOEC concentrations of 675/1250 and 775/1300 mg/l sulfate at hardness concentrations of 80 and 160 mg/l as CaCO3 for *C*. *dubia* reproduction. The ability of hardness to mitigate toxicity was reduced in the 320 mg/l CaCO3 treatment of the study with NOEC/LOECs for sulfate exposure lower at the highest hardness concentration. Lasier and Hardin [20] reported NOEC/LOEC concentration values of 1000/1250 mg/l sulfate in water with moderate-hardness and moderate alkalinity. In testing of a saline mine discharge with the cladoceran, *Moinodapnia macleayi*, van Dam and others [37] found reproductive impairment evident at 1500 μS/cm in a sodium sulfate test solution. These data, and evaluation of Fig 7, would suggest that sulfate levels evaluated in the current study may be reducing neonate production and are approaching the range where toxicity (indicated by traditional endpoints) may occur.

This evaluation demonstrates that streams receiving mining discharges are not consistently toxic to *C*. *dubia* and that site-specific water chemistry characteristics are critical in determining potential toxicity. Although elevated concentrations of dissolved solids did not consistently result in *C*. *dubia* impairment, there was a significantly increased frequency of a toxic response at elevated conductivities. This may indicate the substantial effects of mitigating factors which can reduce toxicity. As indicated, mitigation factors which may play a role in the inconsistent toxicity include: the presence of multiple ions [14, 17, 18], the relatively low toxicity of the dominant sulfate ion [14], and the presence of calcium and magnesium ions [16, 17, 19]. The lack of toxicity to *C*. *dubia* and the apparent effects of mining on macroinvertebrate communities [7, 25] suggest that either the surrogate test organism is not adequately protective of the native taxa or that other factors are contributing (perhaps partially) to community impairment. Potential cumulative or correlated stressors include suspended solids or low levels of metals which may be present in mining discharges [2] and changes in reach-scale geomorphic characteristics [38]. Since conductivity is a general indicator of disturbance [22], many additional stressors potentially occur in the watersheds where samples are collected which could be contributing to the intermittent toxicity and the reduction in neonate production at the highest conductivity levels. Specific mechanisms contributing to impairment must be identified for the purpose of protection and restoration of watersheds influence by mining and other disturbances. The management of water quality alone may be ineffective in protecting and restoring aquatic ecosystems if correlated or cumulative stressors are not considered.
